# Nanoparticle Delivery of Artesunate Enhances the Anti-tumor Efficiency by Activating Mitochondria-Mediated Cell Apoptosis

**DOI:** 10.1186/s11671-017-2169-7

**Published:** 2017-06-12

**Authors:** Rui Liu, Xiwei Yu, Chang Su, Yijie Shi, Liang Zhao

**Affiliations:** 1 School of Pharmacy, Jinzhou Medical University, Jinzhou, 121000 Liaoning People’s Republic of China; 2 School of Veterinary Medicine, Jinzhou Medical University, Jinzhou, 121000 Liaoning People’s Republic of China

**Keywords:** Artemisinin, Oncosis, Apoptosis, Nanoparticles, Mitochondrial

## Abstract

Artemisinin and its derivatives were considered to exert a broad spectrum of anti-cancer activities, and they induced significant anti-cancer effects in tumor cells. Artemisinin and its derivatives could be absorbed quickly, and they were widely distributed, selectively killing tumor cells. Since low concentrations of artesunate primarily depended on oncosis to induce cell death in tumor cells, its anti-tumor effects were undesirable and limited. To obtain better anti-tumor effects, in this study, we took advantage of a new nanotechnology to design novel artesunate-loaded bovine serum albumin nanoparticles to achieve the mitochondrial accumulation of artesunate and induce mitochondrial-mediated apoptosis. The results showed that when compared with free artesunate’s reliance on oncotic death, artesunate-loaded bovine serum albumin nanoparticles showed higher cytotoxicity and their significant apoptotic effects were induced through the distribution of artesunate in the mitochondria. This finding indicated that artesunate-loaded bovine serum albumin nanoparticles damaged the mitochondrial integrity and activated mitochondrial-mediated cell apoptosis by upregulating apoptosis-related proteins and facilitating the rapid release of cytochrome C.

## Background

Artemisinin and its derivatives have been widely used in the treatment of malaria due to their high anti-malarial activity and low toxicity. Researchers also found that artemisinin and its derivatives demonstrated significant anti-tumor activity in virtue of their few toxic side effects and greater tolerance by patients [1]. It was reported that artesunate (Ats) definitely inhibited tumor cell growth and it further induced significant anti-cancer effects in tumor cells [2–4]. Some experiments indicated that Ats caused different degrees of apoptosis and oncosis in tumor cells after 48 h, and that the degrees of apoptosis and oncosis were dependent on the dose of Ats. At low concentrations, Ats did not induce obvious apoptosis in tumor cells and Ats-induced cell death was accompanied by oncosis-like death [5–8]. In order to obtain greater anti-tumor effects, a higher dosage of Ats was applied, but this further confirmed its serious toxicity and bone marrow suppression. Therefore, it is necessary to find an effective treatment to reduce the effective dosage of Ats to enhance its anti-tumor efficiency [9–11]. It was found that the mitochondria played an important role in regulating the apoptotic and oncotic effects of Ats. The mitochondria was also involved in regulating the transduction process of a wide variety of apoptotic signals [12–17]. When the mitochondria was attacked by drugs, its permeability was enhanced and membrane potential had been decreased, thus leading to endometrial swelling of mitochondrial membrane and the rapid release of cytochrome C from the mitochondria into the cytoplasm [18–20]. Furthermore, some proteins from the caspase family were activated, and the cascade reaction of cell apoptosis was induced.

To enhance the anti-tumor effects of Ats, many new techniques were attempted to increase the drug’s distribution in tumor cells or to improve the targeted delivery of drugs into cell organelles to induce cell death [21–23]. Nanoparticles (NPs) as a key tool in targeted cancer treatment have been widely investigated, and they have shown promising potential. As NPs featured a smaller particle size and a high surface area, they could enter the blood circulation via the capillaries and pass through the endothelial cell gap and migrate to the tumor site, thus achieving a drug-targeted distribution and enhancing the bioavailability of the drug. Moreover, NPs could control the release of the drug through the degradation of biomaterial in a long and smooth pattern, ultimately prolonging the eliminating half-life, improving the effective blood concentration, and reducing the dosing frequency. Most of all, drug-loaded NPs could be delivered to specific locations within the cells, improving the treatment efficacy [24–26].

To enhance the anti-tumor effects of Ats at low concentrations, we tried to design novel Ats-loaded bovine serum albumin (BSA) NPs. Because of the low pH in the tumor cells, the accumulation of a large number of hydrogen proton present on the outer mitochondrial membrane or in the intermembrane space, oppositely, the inter mitochondrial membrane is rich in negative charge due to its chemical composition and mitochondria matrix secretion, that makes a electropositive outside and negative inside transmembrane potential which can make a favoring delivery of BSA. Then, the massive accumulation of Ats in mitochondria could effectively trigger mitochondria-mediated apoptosis. The results showed that when compared with the typical oncotic death induced by free Ats, Ats was specifically transferred into the mitochondria with the mediation of BSA NPs and promoted the mitochondria-mediated activation of apoptosis-related caspase proteins. This ignited significant cell apoptosis, thus highlighting the higher cytotoxicity.

## Methods

### Materials

BSA was purchased from Sigma-Aldrich Co. (St Louis, MO, USA), and Ats was purchased from the Guilin Pharmaceutical Corporation (Guilin, People’s Republic of China). SMMC-7721 cells and Plc cells were purchased from the Institute of Biochemistry and Cell Biology of the Chinese Academy of Sciences (Shanghai, People’s Republic of China). All of the other purchased chemicals were of analytical grade; they were obtained from a variety of vendors.

### Preparation and Characterization of Ats-Loaded BSA NPs

According to the previously reported literature [27], Ats-loaded BSA NPs were prepared via a desolvation method. Briefly, Ats-loaded BSA NPs were prepared by quickly dropping 1.0 mL of anhydrous alcohol containing a certain amount of Ats into 0.5 mL of BSA solution at 37 °C until opalescence. With the removal of ethanol by rotary evaporation, Ats-loaded BSA NPs were further precipitated from the medium, and then 8% glutaraldehyde in water (0.5 μL/mg of BSA) was added to induce particle crosslinking under stirring of the suspension over a period of 24 h. Finally, NPs were collected and washed three times with deionized water to further analyze their physical characterizations, including their hydrodynamic diameter, polydispersity index (PDI), zeta potential, and morphology using a Brookhaven Zetasizer (Brookhaven Instruments Corporation, Holtsville, NY, USA) and a transmission electron microscope (JEM-1200EX; JEOL, Tokyo, Japan). Determination of the encapsulation efficiency of Ats in BSA NPs was estimated using a previously reported method [27].

### MTT Assay

Two kinds of tumor cell lines, SMMC-7721 cells and Plc cells, were separately incubated with 20% fetal bovine serum (FBS). The cell growth density was adjusted to l × l0^6^ cells/mL by cell count, and then the cell suspensions were diluted to l × l0^5^ cells/mL. The diluted suspensions were further separately added into a 96-well plate (100 μL per well, about 1 × 10^4^ cells/well) for continuous incubation for 24 h at 37 °C under conditions of 5% CO_2_ and 95% O_2_. The medium was replaced by serum-free medium in the presence of either free Ats or Ats-loaded BSA NPs featuring different concentrations of Ats, and it was subsequently incubated for 24 h. A total of 50 μL of 3-(4,5-dimethylthiazol-2-yl)-2,5-diphenyl tetrazolium bromide (MTT) (5 mg/mL) was added to each well and incubated for 4 h for culture termination. When the tetrazolium dye MTT was reduced to its insoluble formazan, 96-well plates were centrifuged at 1000 rpm for 5 min, and the supernatant was decanted from each well, followed by the addition of 150 μL of dimethyl sulfoxide (DMSO), which completely dissolved the crystals. The absorbance of the solution was measured using a microplate reader (Syneray-2; BioTek Instruments, Inc., Winooski, VT, USA) at 490 nm.

### Intracellular Distribution of the BSA NP Group in Cells

SMMC-7721 cells and Plc cells at the logarithmic phase were selected and treated with trypsin digestion; the cell concentration was adjusted to l × l0^6^ cells/mL. Next, the cultured cells were added into a 6-well cell culture plate for adherence, and the culture medium was removed followed by the addition of rhodamine B-labeled BSA NPs. The nucleus was stained with Hoechst (blue) for 15 min at 37 °C, and the mitochondria was stained by Mitotracker Green FM. The location of BSA NPs in cells was tracked within the cells using confocal laser scanning microscopy (FluoView FV10i; Olympus Corporation, Tokyo, Japan).

### Mitochondrial Membrane Potential Change

JC-1 can be used to determine changes in the mitochondrial membrane potential. When the mitochondrial membrane potential was high, JC-1 was able to freely pass through the cell membrane and formed aggregates within the mitochondria, exhibiting a red fluorescence (excitation wavelength, 525 nm; emission wavelength, 590 nm); when the mitochondrial membrane potential was decreased, JC-1 was transferred from the mitochondrial matrix to the cell cytoplasm to form a green fluorescent monomer (excitation wavelength, 490 nm; emission wavelength, 530 nm). SMMC-7721 cells and Plc cells were respectively seeded in confocal dishes to reach a density of l × l0^6^ cells/mL for continuous incubation for 12 h. Next, the culture medium was discarded and serum-free culture medium containing the dispersion of Ats or Ats-loaded BSA NPs was added into the dish. After 9 h, the medium was discarded and the cells were washed twice with PBS, followed by the addition of 2 mL of JC-1 at a concentration of 2 μmol/L; the cells were then incubated for 30 min at 37 °C under dark conditions. A laser scanning confocal microscope (FluoView FV10i; Olympus Corporation) was used to observe the imaging changes in the mitochondrial membrane.

### ROS Production Measurement and Staining of the Endoplasmic Reticulum (ER)

Cells were incubated with 20% FBS and the cell growth density was adjusted to l × l0^6^ cells/mL by cell count; and then the cell suspensions were diluted to l × l0^5^ cells/mL. The diluted suspensions were further added into 96-well plates (100 μL per well, about 1 × 10^4^ cells/well) for continuous incubation for 24 h at 37 °C under 5% CO_2_ and 95% O_2_. Secondly, free Ats and Ats-loaded BSA NPs were incubated with the cells for 6, 12, and 24 h, followed by continuous incubation with 10 μM of 2,7-dichlorofluorescein diacetate (DCFH-DA; Sigma-Aldrich Co.) for about 30 min. Ice-cold PBS buffer was used to wash the cells three times to remove the uninternalized NPs. The intracellular DCF fluorescence intensity, which is excited at 485 nm and emitted at 530 nm, was detected using a microplate reader (Synergy-2; BioTek Instruments) to investigate the extent of oxidative stress. The test groups were treated with SMMC-7721 cells and Plc cells for 24 h, and the ER-Tracker Blue–White DPX probe (Molecular Probes, Eugene, OR, USA) was added into the cells for incubation for 30 min. After discarding the loading solution and washing the cells with PBS, the morphology change of the ER was observed by confocal laser scanning microscopy.

### Cell Oncosis and Apoptosis Evaluation by Flow Cytometry

According to the protocol of our previous study [28], an Annexin V–fluorescein isothiocyanate (FITC)/propidium iodide (PI) staining assay was used to evaluate the cell oncosis and apoptosis induced by free Ats and Ats-loaded BSA NPs. Cells were lysed with typsin and seeded into six-well plates at a concentration of l × l0^6^ cells/mL for 24 h of continuous incubation. Next, the culture medium was removed and serum-free medium containing free Ats and Ats-loaded BSA NPs was added into the wells. After treatment, the cells were collected and suspended in Nicoletti buffer (Beijing 4A Biotech Co., Ltd., Beijing, People’s Republic of China) containing PI- and FITC-labeled Annexin V (AV-FITC). The morphological change of the cells was observed by confocal laser scanning microscopy. To verify the cell apoptosis and oncosis rates induced by the Ats-loaded NPs, the percentages of early apoptotic (Q4), oncotic (Q2), necrotic (Q1), and live cells (Q3) were quantified by flow cytometry.

### Western Blot Analysis of Apoptosis-Related Proteins and Cytochrome C in Cells

A western blot assay was performed to determine the levels of relative proteins when free Ats or Ats-loaded NPs were incubated with SMMC-7721 cells for 24 h. Cells were lysed with ice-cold radioimmunoprecipitation assay (RIPA) buffer containing a protease inhibitor cocktail and phosphatase inhibitors (Roche, Basel, Switzerland). Protein concentrations were determined using a modified BSA assay kit (Thermo Fisher Scientific, Waltham, MA, USA) and normalized before loading on 10% sodium dodecyl sulfate (SDS)–polyacrylamide gel electrophoresis (PAGE). The levels of the targeted proteins were photographed and analyzed using a UVP gel analysis system (iBox Scientia 600; UVP, LLC., Upland, CA, USA).

## Results

### Characteristics of Ats-Loaded BSA NPs and Cellular Viability Study

**Fig. 1:**
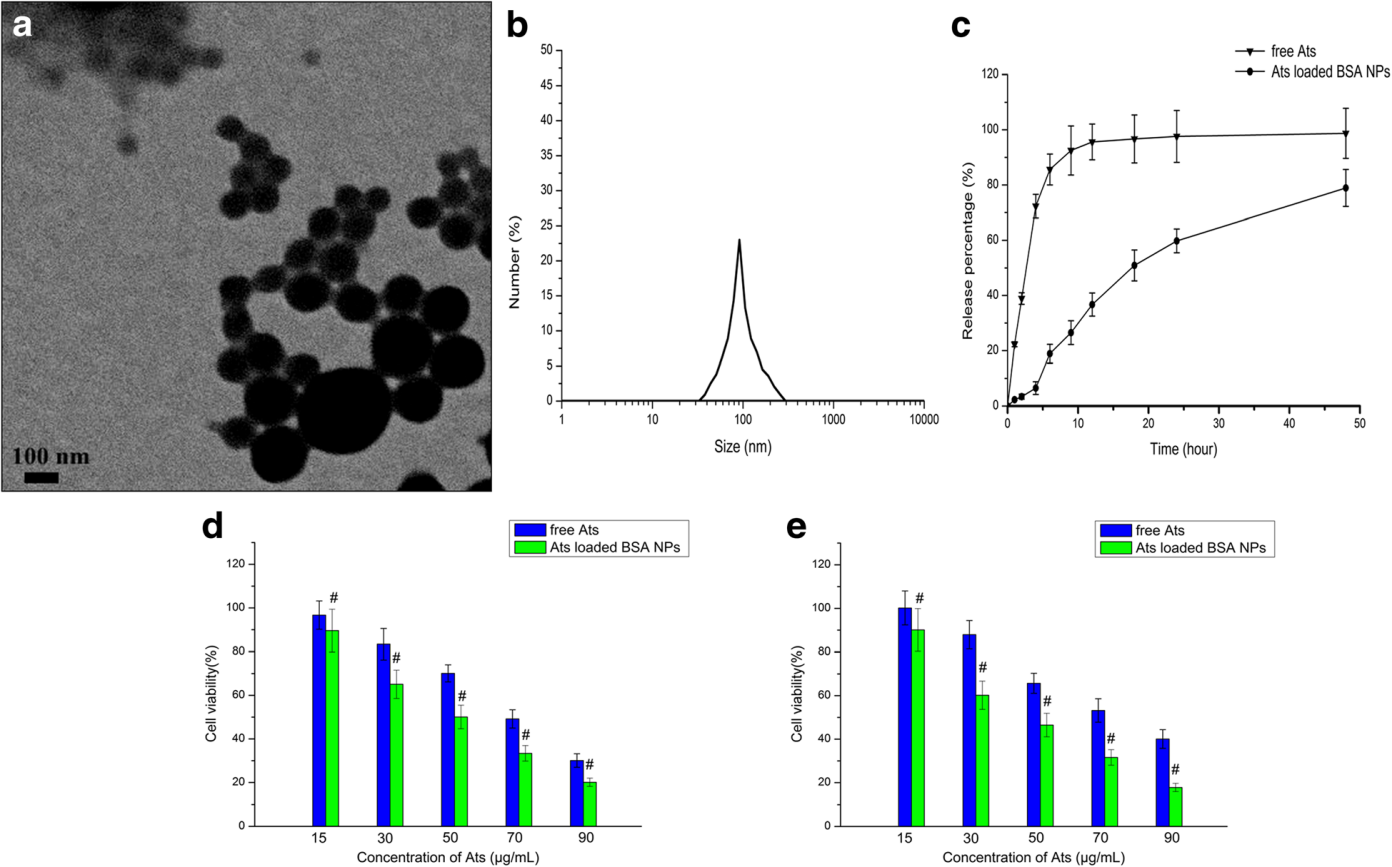
Characterization of Ats-loaded BSA NPs. **a** TEM image of Ats-loaded BSA NPs. **b** Dynamic light scattering (DLS) analysis of the obtained Ats-loaded BSA NPs. **c** The in vitro release profile of Ats-loaded BSA NPs in phosphate-buffered saline with a pH of 7.4 at 37 °C for 48 h. Viability of SMMC-7721 cells (**d**) and Plc cells (**e**) following incubation with different amounts of free Ats and Ats-loaded BSA NPs for 24 h. The data are presented as the mean ± SD (*n* = 3). ^#^ *P* < 0.05 versus the corresponding free Ats

It was observed in Fig. 1a, b that Ats-loaded BSA NPs showed a spherical shape, and they were homogenously dispersed with a lower PDI at 0.016. The average particle size of Ats-loaded BSA NPs was about 99.9 ± 2.3 nm and the zeta potential was negative and valued at around –25.6 ± 4.3 mV. The release profile showed a smooth and sustained release which was shown in Fig. 1c. Compared with the rapid release of free Ats in medium in vitro, Ats entrapped in the core of BSA NPs was slowly diffused from the interior of NPs into the medium and showed a smooth and sustained release pattern, owing to the continuous degradation of BSA. More than 85% of free Ats was released completely within the first 6 h, while the total accumulative amount of drug released from the NPs into the media within a 48-h period was 78.9%. This indicated that NPs could control the release of the drug via the degradation of biomaterials in a long and smooth pattern, thus prolonging the elimination half-life, improving the effective blood concentration, and reducing the dosing frequency.

MTT was used to examine the inhibiting effects of free Ats and Ats-loaded BSA NPs in SMMC-7721 cells and Plc cells at different time intervals. The results (Fig. 1d, e) showed that the cytotoxicity of free Ats increased with the increase of the drug concentration, and Ats-loaded BSA NPs showed the gradual enhanced cytotoxicity. This proved that Ats and Ats-loaded BSA NPs inhibited the growth of tumor cells and that the inhibition ratio was dependent on the dose of Ats. Compared with free Ats, Ats-loaded BSA NPs demonstrated higher cytotoxicity and higher sensitivity in both cells, and they resulted in greater cell inhibition. As shown in Fig. 1d, e, treatment of both cells with Ats-loaded BSA NPs caused a significant decrease in cell viability at 24 h when compared with that of free Ats. The 50% maximal inhibitory concentration (IC50) values for the SMMC-7721 cells and Plc cells treated with Ats-loaded BSA NPs were 50.1 and 44.9 μg/mL at 24 h, respectively, which is compared with the values obtained of 69.2 and 74.9 μg/mL at 24 h in cells treated with free Ats. This indicated that when Ats was loaded in BSA NPs, it might change its intracellular location, as mediated by the NPs, and ultimately killed more cells.

### In Vitro Cellular Uptake of BSA NPs

**Fig. 2:**
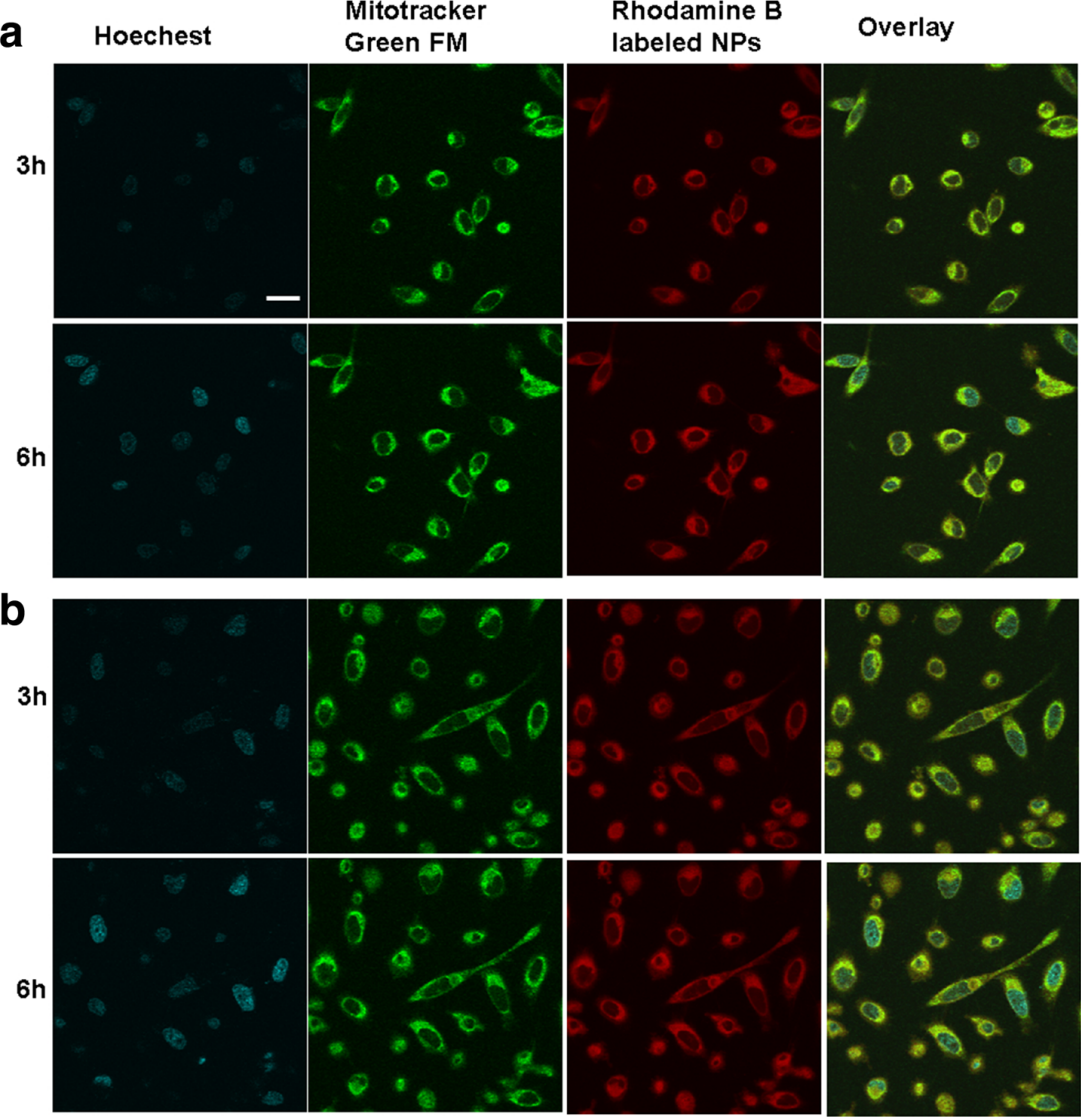
The in vitro cellular distribution of BSA NPs after being incubated with different tumor cells. Fluorescent image of SMMC-7721 cells (**a**) and Plc cells (**b**). *Scale bar*, 100 μm

The intracellular distribution and location of BSA NPs in both types of tumor cells were observed by confocal laser scanning microscopy, as shown in Fig. 2. After rhodamine B-labeled NPs were co-cultured with cells for 3 h, red fluorescence was clearly seen in the cytoplasm; with the passage of time, the majority of BSA NPs were internalized intracellularly and diffused into the cytoplasm, displaying enhanced time-dependent red fluorescence. It was also observed that BSA NPs located in the cytoplasm had been co-located with the mitochondria, as evident by the appearance of yellow fluorescence, which served to indicate that the inherent red fluorescence of rhodamine B-labeled NPs and the green fluorescence emitted by the mitochondrial indicator MitoTracker® green FM had been merged. This proved that the internalized BSA NPs could be specifically accumulated within the mitochondria, highlighting the possibility that Ats could be delivered to the mitochondria with the mediation of BSA NPs.

### Mitochondrial Membrane Potential Analysis

**Fig. 3:**
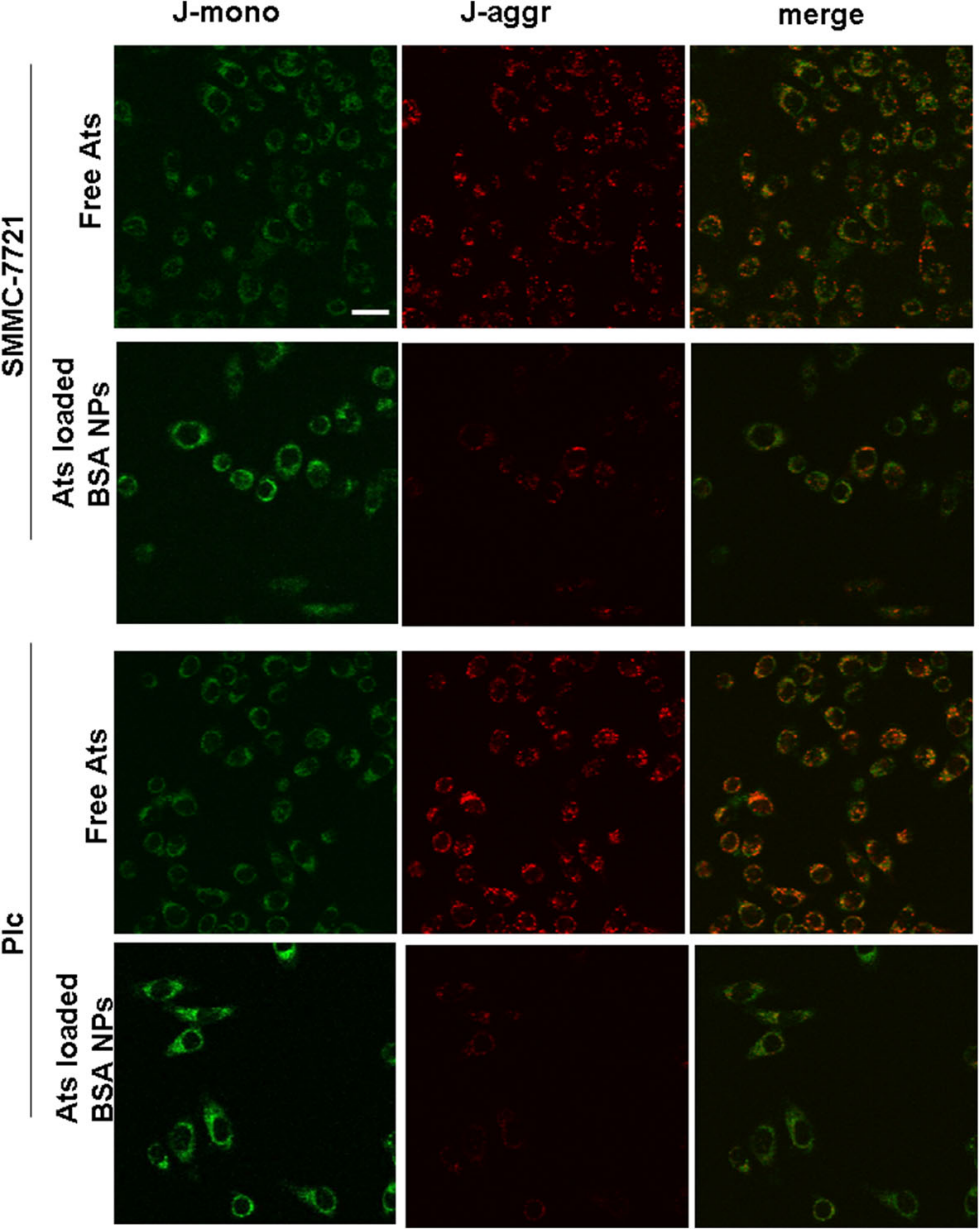
Imaging change of the mitochondrial membrane potential following incubation of free Ats and Ats-loaded BSA NPs with SMMC-7721 cells and Plc cells. *Scale bar*, 100 μm

To clarify whether Ats-loaded BSA NPs interfered with mitochondrial function following the delivery of Ats in the mitochondria, changes in the mitochondrial membrane potential were determined. Figure 3 demonstrated that after JC-1 staining, the majority of the mitochondria in tumor cells treated with free Ats exhibited strong red fluorescence and weak green fluorescence intensity. This suggested that the majority of JC-1 existed in an aggregated state, reinforcing the integrity of the mitochondrial membrane and a higher potential. On the contrary, when JC-1 stained the Ats-loaded BSA NPs-treated cells, the mitochondria in both tumor cells exhibited stronger green fluorescence, indicating that the mitochondrial membrane was seriously damaged and its potential was significantly decreased. Taken together, it proved that Ats was successfully delivered to the mitochondria with the mediation of BSA NPs, resulting in mitochondrial membrane depolarization.

### ROS Production Measurement and Staining of the ER

It was widely confirmed that the generation of a large number of ROS can cause phospholipid peroxidation in the inner mitochondrial membrane and that it can also induce a decrease in the mitochondrial membrane potential, thus resulting in the rapid release of cytochrome C. We used DCFH-DA as a fluorescent probe to detect the change of ROS. DCFH-DA passed freely through the cell membrane into the cell and was transformed into DCFH by esterase hydrolysis. The generated DCFH cannot pass through the cell membrane, and it can be easily loaded into the cells. Intracellular ROS oxidized non-fluorescent DCFH to DCF with a green fluorescent color. Therefore, DCF fluorescence detection can indicate the level of intracellular ROS.

**Fig. 4:**
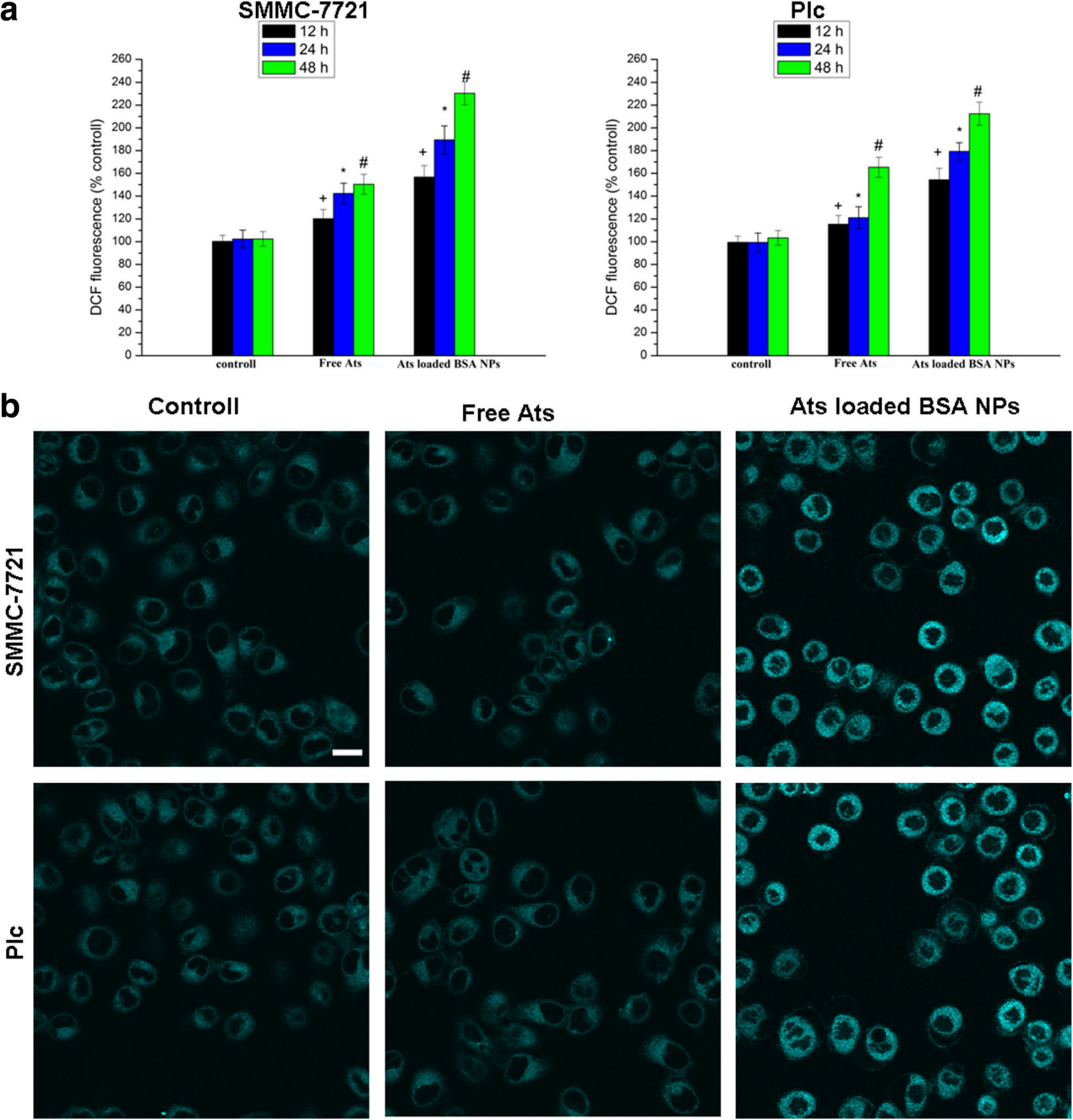
Quantification of ROS generation in cells treated with free Ats and Ats-loaded BSA NPs at different times (**a**). ER staining with the ER-Tracker Blue–White DPX probe (**b**). *Scale bar*, 100 μm. The data are presented as the mean ± SD (*n* = 3). ^+^ *P* < 0.05 versus the control group at 12 h, *P < 0.05 versus the control group at 24 h, ^#^ *P* < 0.05 versus the control group at 24 h

When both cells were treated with free Ats and Ats-loaded BSA NPs for a certain period of time, the amount of intracellular ROS had been also increased, showing a time-dependent relationship. Compared with free Ats, the generation of ROS in SMMC-7721cells and Plc cells treated with Ats-loaded BSA NPs was significantly enhanced. Figures 4a demonstrated that the ROS levels in SMMC-7721 cells and Plc cells exposed to Ats-loaded BSA NPs for 48 h had been increased to 1.53-fold and 1.28-fold, respectively, when compared with SMMC-7721 cells and Plc cells treated with free Ats. This supported the idea that the NPs accelerated the production of intracellular ROS. Compared with the control group and free Ats, and after being treated with Ats-loaded BSA NPs, the fluorescence staining intensity from the ER-Tracker Blue–White DPX as an ER-specific dye was significantly increased, suggesting that ER stress was also triggered in the Ats-loaded NPs-treated cells with a corresponding increase in the ROS level. This finding highlighted that Ats was specifically located in the mitochondria, as mediated by BSA NPs; this led to a significant increase in the level of oxygen-free radicals within the cells, thus triggering the induction of ER stress and activating the mitochondrial pathway to induce caspase-dependent cellular apoptosis.

### Evaluation of Cell Apoptosis and Necrosis

**Fig. 5:**
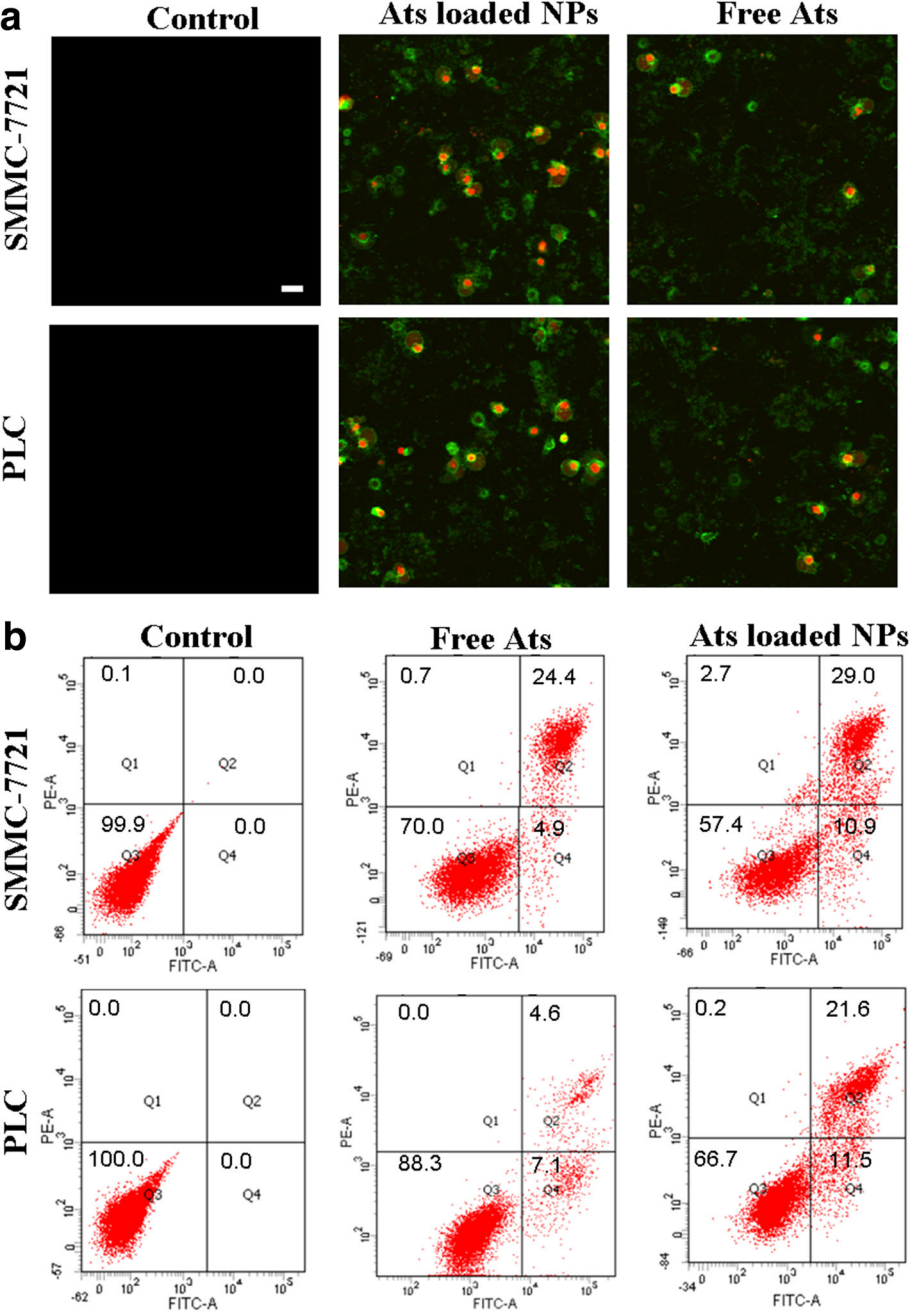
Morphology of the ultrastructural changes of cells treated with free Ats and Ats-loaded BSA NPs using Annexin V-FITC/PI staining assay (**a**). *Scale bar*, 100 μm. Flow cytometer analysis of the cellular apoptosis and oncosis after 24 h of incubation with the free Ats and Ats-loaded BSA NPs, respectively (**b**)

Cells were treated by an Annexin V-FITC/PI staining assay. Living cells did not bind to Annexin V-FITC/PI, thus no fluorescence appeared. Apoptotic cells did not bind to PI, but they were stained with Annexin V-FITC, yielding green fluorescence. On the contrary, for the oncotic cells, their cell membranes were damaged to some extent, and the cell nuclei were dilated to break down into pieces, thus showing both green and red fluorescence. As shown in Fig. 5a, compared with the control group, when free Ats and Ats-loaded NPs were incubated with cells for 24 h, strong green and red fluorescence were observed in the cells, indicating that free Ats and Ats-loaded BSA NPs induced tumor cell oncosis and apoptosis. Especially after being treated with Ats-loaded BSA NPs, the staining fluorescence intensities obtained from Annexin V-FITC and PI had been significantly increased, suggesting that the degrees of oncosis and apoptosis were significantly enhanced in the Ats-loaded NPs-treated cells.

The percentages of early apoptotic (Q4), oncotic (Q2), necrotic (Q1), and live cells (Q3) were shown in Fig. 5b. This finding demonstrated that when cells were treated with free Ats, the oncotic rates were gradually increased to 24.4 and 4.6%, and the apoptotic rate remained at 4.9 and 7.1% in SMMC-7721 cells and Plc cells, respectively, suggesting that free Ats triggered the occurrence of oncosis and apoptosis to lead to cell death. On the contrary, Ats-loaded BSA NPs significantly improved the rate of cell apoptosis and oncosis. The apoptotic ratios were significantly increased to 10.9% in SMMC-7721 cells and to 11.5% in Plc cells. The oncotic ratios were increased to 29.0% in SMMC-7721 cells and to 21.6% in Plc cells. This indicated that the mitochondrial delivery of Ats with the mediation of BSA NPs accelerated the death of tumor cells by enhancing the oncotic and apoptotic effects. Ats-loaded BSA NPs triggered the apoptotic signal transduction process and promoted the mitochondrial-mediated cascade reaction of cellular apoptosis.

### Western Blot Analysis

**Fig. 6:**
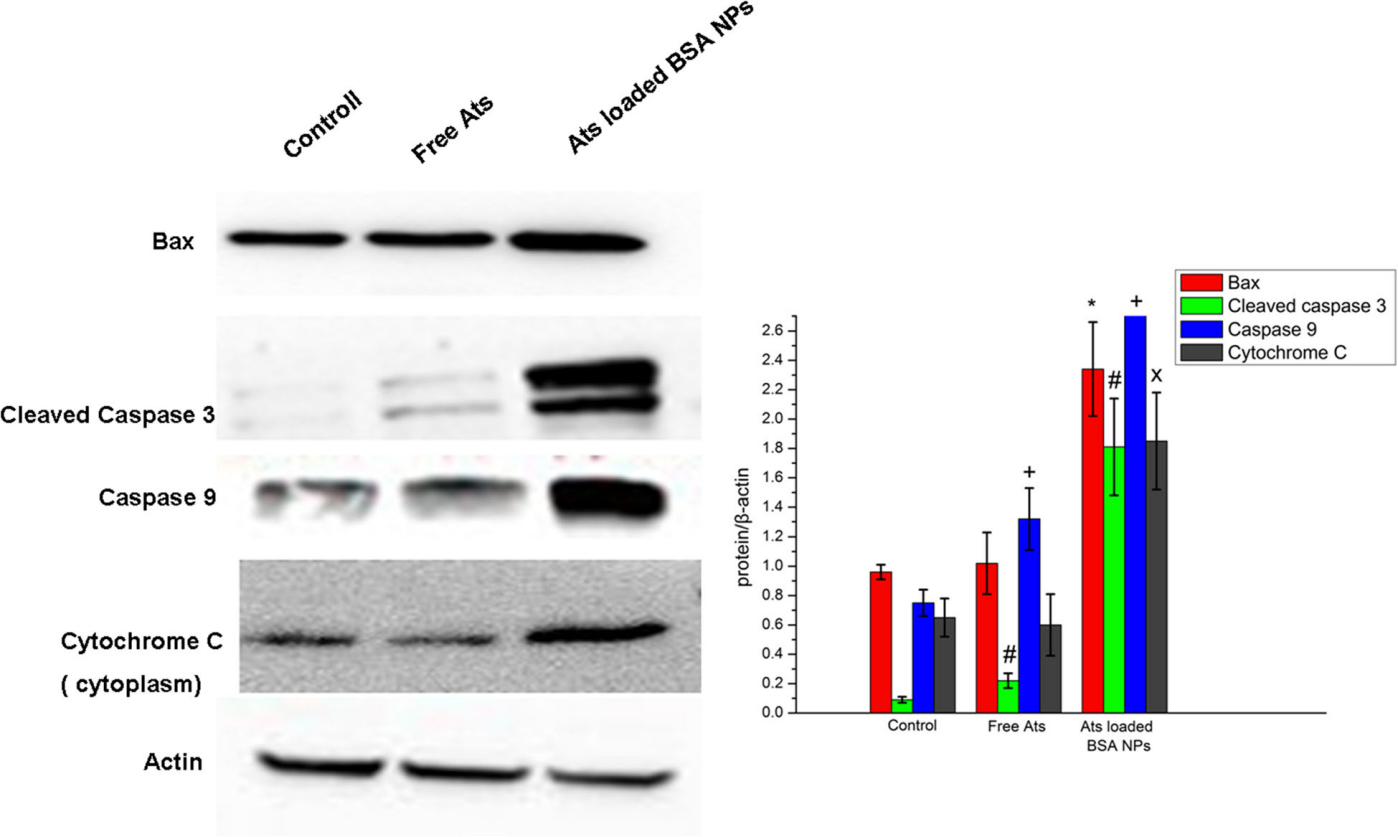
Western blot analyses of the expression levels of cleaved caspase-3, caspase-9, Bax, and cytochrome C in SMMC-7721 cells. *P < 0.05 versus the Bax protein expression of the control group; ^#^ *P* < 0.05 versus the cleaved caspase-3 expression of the control group; ^+^ *P* < 0.05 versus the caspase-9 protein expression of the control group; ^x^ *P* < 0.05 versus the cytochrome C protein expression of the control group. Data were presented as the mean ± SD (*n* = 3)

To explore the dependence of cell death on the apoptosis induced by free Ats and Ats-loaded NPs, a western blot assay was performed to detect the expression of apoptosis proteins. It was found that in Ats-loaded NPs-treated SMMC-7721 cells, the intracellular expression level of the Bax protein was significant increased (Fig. 6). This finding suggested that with the help of BSA NPs, Ats was accumulated in the mitochondria and caused mitochondrial dysfunction. The cytoplasmic Bax monomer protein was transferred to the mitochondria’s outer membrane and underwent oligomerization, forming a protein channel in the mitochondria’s outer membrane, thus further leading to an increase in membrane permeability. The expression level of cytochrome C in the cytoplasm was also particularly and significantly enhanced, and the expressions of caspase-3 and caspase-9 were found to demonstrate an upward trend. Therefore, owing to the higher membrane permeability of the mitochondria, cytochrome C was rapidly released into the cytoplasm, activating cell death-signaling proteins (caspases) and promoting the cascade reaction of cellular apoptosis. In contrast, free Ats had no significant difference on the expression of apoptosis-related proteins and cytochrome C, suggesting that free Ats did not trigger mitochondria-mediated cell apoptosis and it primarily relied on oncosis to lead to cell death. BSA NPs enhanced the drug’s accumulation in the mitochondria and activated mitochondrial-mediated apoptotic effects, thus leading to significant apoptosis and increasing the expressions of the primary apoptosis-relevant proteins, as shown in our western blot analyses.

## Discussion

Oncosis and apoptosis represent the two different ways in which cells undergo death. Apoptosis is an active process of programmed cell death that occurs in multicellular organisms. Oncosis, on the other hand, describes a caspase-independent cell death that is characterized by swelling, increased permeability, and membrane rupture, which is often referred to as necrosis. This form of cell death is believed to be accidental and uncontrolled. Based on our investigation, we found that Ats inhibited the growth of tumor cells and that the inhibition ratio was dependent on the dose of Ats. Ats primarily depended on the degree of oncosis and led to cell death; it also activated caspase-independent cell death in the form of oncosis. Conversely, and separately from the occurrence of obvious oncosis-like death, when tumor cells were treated with Ats-loaded BSA NPs, Ats-loaded BSA NPs were internalized into the cytoplasm and were quickly located within the mitochondria to release Ats, as mediated by the NPs. Ats in the mitochondria generated ROS and triggered ER stress; it further activated the mitochondria-meditated caspase-dependent cell apoptotic pathway by reducing the mitochondrial membrane potential, releasing cytochrome C, and promoting the protein expressions of Bax, cleaved caspase 3, and caspase 9. Taken together, Ats-loaded BSA NPs increased the mitochondrial delivery of Ats and enhanced the degree of oncosis and apoptosis to induce cell death, thus increasing the cytotoxicity of the drug and inducing significant cell death.

## Conclusions

Briefly, we clarified that free Ats in the tumor cells was strongly dependent on the degree of oncosis to inhibit the proliferation of tumor cells in the form of an oncosis-like death; thus, the cytotoxicity of the drug was limited and undesirable. In contrast, Ats-loaded BSA NPs activated the mitochondrial apoptotic pathway and simultaneously triggered oncotic effects; together, they enhanced the synergistic anti-tumor efficacy of Ats. The results of this study highlighted the significance of Ats-loaded BSA NPs in the enhancement of the cytotoxic and apoptotic effects of Ats, and they further signify the role of BSA NPs in diversifying the pathways of cell death induced by Ats. Compared with free Ats, Ats-loaded BSA NPs induced greater cytotoxicity and significant cell apoptosis effects in tumor cells.
